# Clinical perspective on innovative insulin delivery technologies in diabetes management

**DOI:** 10.3389/fendo.2024.1308319

**Published:** 2024-10-01

**Authors:** Güvenç Koçkaya, Tadej Battelino, Goran Petrovski, Johan Jendle, Beatrix Sármán, Nancy Elbarbary, Damla Gökşen, Mohammed Alharbi, Birol Tibet, Amir Mustapha Sharaf, Selin Ökçün, Filiz Öztürk, Mustafa Kurnaz

**Affiliations:** ^1^ Department of Health Economics and Outcome Research, ECONiX Research, Samsun, Türkiye; ^2^ Department of Pediatric and Adolescent Endocrinology, University Medical Center Ljubljana, Ljubljana, Slovenia; ^3^ Sidra Medicine, Weill Cornell, Ar-Rayyan, Qatar; ^4^ Medical Sciences, Örebro University, Örebro, Sweden; ^5^ Department of Medicine, Faculty of Medicine Budapest Hungary, Semmelweis University, Budapest, Hungary; ^6^ Department of Pediatrics, Ain Shams University, Cairo, Egypt; ^7^ Department of Pediatrics, Faculty of Medicine, Ege University, Bayraklı, İzmir, Türkiye; ^8^ Deparment of Information Secuity Operation, Ministry of Health Saudi Arabia, Riyadh, Saudi Arabia; ^9^ Health Economics and Outcomes Research, ECONiX Research, Tunis, Tunisia

**Keywords:** diabetes mellitus, diabetes management, multiple daily injections (MDI), continuous glucose monitor (CGM), smart insulin pens, new technologies

## Abstract

**Introduction:**

The primary objective of this study is to report the results of an online questionnaire and the in-person discussion sessions of physicians specializing in diabetes care in which their opinions about current diabetes management was obtained.

**Methods:**

The Diabetes Innovation Summit 2023 drew attendance from a diverse group of specialized physicians from multiple countries. A comprehensive literature review was conducted to examine the technologies and medical needs associated with diabetes management. Using the results of the review, a questionnaire was developed by three experts from the steering committee to solicit feedback from specialized physicians. The online survey was made accessible between 10th December 2022 and 10th January 2023. Following the online survey, six structured in-person discussion sessions were conducted with specialized physicians from the Middle East, Central-Eastern Europe, and North Africa regions.

**Results:**

The study revealed that about 59% of survey requests were answered, with many participants being pediatric endocrinologists from North Africa. Around 60% of diabetes patients followed Multiple Daily Injections (MDI) according to specialized physicians. Among MDI users, 62% employed Blood Glucose Monitors (BGM), 31% used intermittent-scanning Continuous Glucose Monitors (isCGM), and 23% used CGM. In North Africa, nearly 90% of patients used MDI due to financial constraints. While physicians focused on both Time in Range (TIR) and HbA1c for MDI-treated patients, satisfaction with TIR achieved was expressed by 31%, while 74·1% believed Real-Time CGM (rtCGM) was effective. Concerns arose about potentially misleading HbA1c results and the relatively low patient achievement of target TIR despite CGM usage. The Smart MDI System was seen favorably compared to other applications. The system’s affordability was a significant barrier, particularly in the Middle East and Africa.

**Conclusion:**

The present study highlights that physicians are generally supportive of utilizing new technology. The questionnaires and the open discussion revealed the expectation that the Smart MDI technology provides better control, primarily by identifying missed boluses, while expressing concerns on the use of the technology by teenagers and children, who might forget the device and be reluctant to use in public, and by the older population, who might be challenged by the technology.

## Introduction

Diabetes mellitus is a chronic condition caused by either a lack of insulin production (T1D) or insulin resistance (T2D), leading to elevated blood glucose levels. This results in complications like blindness, renal failure, dementia, amputations, and cardiovascular events, ultimately causing premature death. In 2021, 537 million adults globally had diabetes, with an additional 240 million undiagnosed cases. Three-quarters of these individuals live in low- and middle-income countries (1). In 2021, there were approximately 8·4 million individuals with T1D worldwide: 1·5 million (18%) of them aged below 20 years, 5·4 million (64%) were 20-59 years and 1·6 million (19%) were 60 years or older (2). Epidemiologic studies show that there is a 3% increase in age-standardized death rate from diabetes during the period 2000 and 2019. The death rate was even greater (13%) in low-to-middle income countries (3). Not only is it associated with increased mortality and complications, but it also consumes a large percentage of health care expenditures. Overall, 9% of total health expenditures (USD 966 billion) were related to diabetes (1).

The main goals of diabetes treatment are to provide tight glycemic control, relieve symptoms, reduce diabetes-related mortality and prevent the development of acute complications (severe hypoglycemia and diabetic ketoacidosis) and long-term complications noted above as well as increasing the quality of life of the individuals living with diabetes (4). Overall, there is inadequate glycemic control in diabetic patients especially those who require insulin for their treatment (5). Aids to diabetes management include frequent blood glucose monitoring (BGM) by fingerstick or by continuous glucose monitoring (CGM) as well as access to a well-functioning healthcare infrastructure (6). Diabetes patients can choose between BGM systems, which measure glucose in capillary blood, and CGM systems, which measure glucose in interstitial fluid. While BGM is the traditional method, CGM use has rapidly increased. CGM systems are either real-time (rtCGM), providing updates every 5 minutes if within range, or intermittently scanned (isCGM/flash), requiring users to scan the sensor for current glucose values on a reader or smartphone (7).

Glycemic control has traditionally been assessed by measurement of glycated hemoglobin (HbA1c). However, over the last 10 years, the use of CGM and the glucose metrics derived from these devices has become the favored method of assessing glycemic control by many if not most healthcare providers working with diabetes. CGM allows the healthcare professionals (HCPs) and diabetic patients to assess the percent of time spent in the target range (TIR=70-180 mg/dL), time below target range (TBR <70 mg/dL and <54 mg/dL) and time above target range (TAR >180 mg/dL and >250 mg/dL), and act upon predictive glucose information (8). It has been shown that starting CGM early after being diagnosed with T1D reduces HbA1c level for young people (9).

The American Diabetes Association (ADA) and the European Association for the Study of Diabetes (EASD) have published guidelines which set out the goals for times-in-ranges (10). The goals for most diabetic patients are: TIR 70-180 mg/dL >70% of the readings; TBR of <70 mg/dL of <4% of the readings and TBR of <54 mg/dL of <1% of the readings; and TAR >180 mg/dL <25% of the readings and TAR >250 mg/dL <5% of the readings (11).

There are several methods of insulin delivery available to diabetic patients. These include the traditional insulin syringe, insulin pens, smart insulin pens (SIPs) and insulin pumps [with or without concomitant CGM and with or without automated insulin delivery (AID)]. While AID systems provide the best glycemic control compared with the other methods, there are many reasons why diabetic patients may not have access to or want such a system. For those diabetic patients, SIPs when combined with CGM and an insulin dosing calculator represent a new category of diabetes management – the smart insulin pen system – for those managing their insulin with multiple daily injections (MDI) and can provide many of the benefits of an AID system. A SIP records the time and amount of the dose of short-acting insulin and transmits this data to an app on a smartphone and thereby providing the dose calculator essential information that takes insulin-on-board into account. This can reduce the risk of insulin stacking and provides a more accurate meal and correction insulin dose. The report that the system generates is invaluable to the HCPs, the diabetic patients and their caregiver because it can show the consequences of behaviors such as missing or late insulin doses and inaccurate carbohydrate counting thus providing an opportunity for targeted diabetes education (12, 13).

Recently developed, the Smart MDI system, which combines a CGM with a SIP, offers a comprehensive solution for individuals managing diabetes through insulin injections. This system aims to reduce the physical and mental burden of diabetes management by integrating smart-enabled technological devices. The Smart MDI includes a CGM, an injection port, and a smart insulin pen, providing advanced tools for individuals with type 1 or type 2 diabetes to better manage their condition.

Adolfsson et al. reported that there was a 43% reduction in missed bolus doses of the participants in the ≥180-day follow-up from the initiation of use of the smart pens in their study. They also found a significant increase in TIR of 1·9 hr./day with reduced time spent for hyperglycemia (>180 mg/dL) and hypoglycemia (<54 mg/dL) (14). Increasing concordance with insulin therapy not only has a positive effect on glycemic control but also potentially reduces healthcare costs and utilization (12). Use of the bolus calculator in the SIP system has been associated with a 0·7-1% reduction in HbA1c level and a reduced fear of hypoglycemia. A SIP system can help patients achieve better glycemic results by reminding the user to take their insulin.

With regard to the published literature, the aim of this study is to capture the opinion of diabetes specialists from several regions to better understand how they currently use and how they may use diabetes technologies in the management of diabetic patients using MDI.

## Methods

### Steering committee

The nominal group technique (NGT) is a widely used method for developing consensus on a given topic. Through NGT, experts provide their input and group consensus is reached through in-person meetings. Within the focus group setting, NGT provides a rigorous approach for acquiring trustworthy and pertinent qualitative information from a group of experts. Through its structured questioning approach, NGT encourages participation from all members, enabling the synthesis of divergent viewpoints on a shared area of interest and identification of consensus areas and priorities for change (15).

Using the nominal group technique (NGT), the steering committee assembled a group of diabetes experts from academia, industry, and consulting backgrounds. The steering committee consists of diabetes clinicians and health economics experts from different countries. The committee developed a structured questionnaire and further led face-to-face discussions to elicit expert opinions.

### Survey design

**Figure 1:**
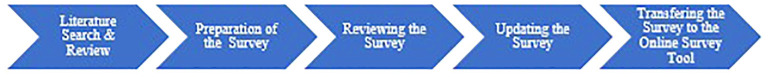
Steps of Questionnaire Preparation.

A comprehensive literature review was conducted to examine the technologies and medical needs associated with diabetes management. Using the results of the review, a questionnaire was developed by three experts from the steering committee to solicit feedback from specialized physicians. The remaining six members of steering committee reviewed the questionnaire. The survey was finalized after the review of other clinicians in the steering committee. The pilot test of the questionnaire was conducted with a randomly selected group of 10 specialized physicians. The specialized physicians randomly selected for the pilot test were experts in the relevant field, ensuring that the feedback received was both informed and constructive. Their responses and feedback were utilized to assess the format, language, and clarity of the specified items. Based on the feedback provided by the specialized physicians, several modifications were made to the questionnaire, including rephrasing ambiguous questions, adding more response options to certain items, and restructuring the survey to improve its logical flow (**Figure 1**).

The questionnaire consisted of five sections and 40 questions in total. The sections were Demographics, General Insights, Expectations, Barriers & Drivers, and New Therapeutic Options. The questionnaire was transferred to an online survey tool and was sent via e-mail to 100 specialized physicians working in Middle East, Eastern Europe, and Africa in order to obtain expert opinions from different geographical regions. Efforts were made to follow up with non-responders to ensure that the invitation to participate was received and understood. The online survey was made accessible between 10th December 2022 and 10th January 2023. The online questionnaire was filled in completely anonymously. Descriptive analyses were conducted using Microsoft Office Excel 360.

### Demographics of the specialized physicians participating in the study

The selection of participants for the study involved identifying specialized physicians across different countries to ensure a diverse and representative sample. In order to avoid any selection bias and increase the generalizability of the findings, all physicians who attended The Diabetes Innovation Summit 2023 event constituted the participants. Volunteers from among the physicians who attended the event participated in the survey. The participants represented a broad demographic, including various age groups, genders, and medical specialties. This diversity was crucial to ensure that the topic was evaluated across different demographic locations.

The Diabetes Innovation Summit 2023 event was held in Istanbul on 13th and 14th of January 2023. The Diabetes Innovation Summit 2023 drew attendance from a diverse group of specialized physicians, including pediatric endocrinologists, adult endocrinologists, diabetologists, internal medicine specialists, and general pediatricians from multiple countries, including Bosnia and Herzegovina, Croatia, Czech Republic, Egypt, Finland, Hungary, Iraq, Latvia, Lebanon, Libya, North Macedonia, Poland, Qatar, Saudi Arabia, Serbia, Slovenia, The United Arab Emirates, The United Kingdom and Türkiye. As part of the study, these experts were divided into small groups based on geographic distribution and engaged in an in-person post-survey discussion session at the Istanbul meeting.

The study adhered to the ethical guidelines set forth by the Declaration of Helsinki. All participating physicians were informed about the study’s objectives, procedures, and their rights as participants. Written informed consent was obtained from each participant, ensuring that they were fully aware of the voluntary nature of their involvement and their right to withdraw from the study at any point without any consequences. The confidentiality and anonymity of the participants were strictly maintained throughout the study, and all collected data were securely stored and accessed only by authorized personnel.

### Discussion session design

Following the online survey, six structured discussion sessions were conducted in person with specialized physicians from the Middle East, Central-Eastern Europe, and North Africa regions. Physicians were organized into distinct groups based on their respective regions and encouraged to provide feedback on the survey results as well as their clinical practices. The sessions were moderated by members of the steering committee.

### Evaluation and reporting

The steering committee members moderated, evaluated, and reported on the discussion sessions. Moderation was tailored to the specific questionnaire results, with each question discussed among specialized physicians from the corresponding region under the guidance of steering committee members. The steering committee members who did not participate in the discussions were responsible for reporting on the sessions.

## Results

### Demographics

We obtained a responses from 59% of requests to complete the survey. The non-participation of the remaining 41% was primarily due to personal preferences and scheduling conflicts, rather than any inherent issues with the survey itself. A significant number of participants work in North Africa, with more than half of the participants being pediatric endocrinologists. A similar number of physicians from the Eastern Europe and Middle East regions participated in the study (**Table 1**).

**Table 1:** Distribution of physicians.

Region	n (%)
North Africa	26 (44·0%)
Central-Eastern Europe	17 (28·8%)
Middle East	13 (22·0%)
Other	3 (5·0%)
Specialty	
Pediatric Endocrinologist	33 (55·9%)
Adult Endocrinologist	13 (22·0%)
Diabetologist	9 (15·2%)
General Paediatrician	2 (3·3%)
Internal Medicine Specialist	1 (1·6%)
Other	1 (1·6%)

**Figure 2:**
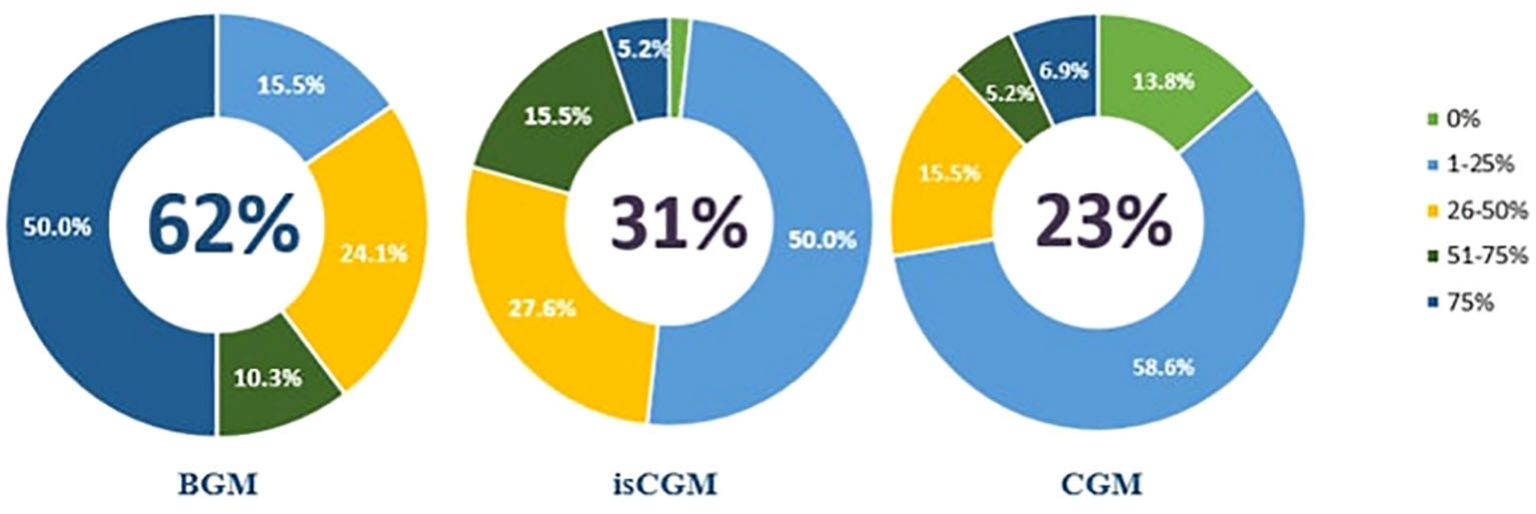
BGM, isCGM and CGM Usage Rates of Diabetic Patients Using MDI.

60% of diabetic patients were using MDI according to the clinical practice of specialized physicians. Specialized physicians stated that among diabetic patients who employ MDI, 62% use BGM, 31% isCGM and 23% CGM (**Figure 2**).

Physicians from North Africa stated that almost 90% of their diabetic patients use MDI because they must pay out of pocket for other treatments and devices which include medication pens, blood glucose meters, strips, injection needles and ketone monitors. In addition, they stated that, their patients used BGM more than CGM. Physicians from Central-Eastern Europe have stated that the usage ratio of MDI in adults is 20 to 30%. For example, MDI is only reimbursed for T1D patients, but not for T2D patients in Czech Republic. This leads to less MDI use.

### The nominal group technique results

#### The following will describe and compare the results of the debated topics of questionnaire with the outcome of face-to-face discussion followed by the shared conclusions

##### Questionnaire: Attitudes and concepts about the use of the glucose metrics HbA1c measurements, TIR and the ability to achieve glycemic goals

**Figure 3:**
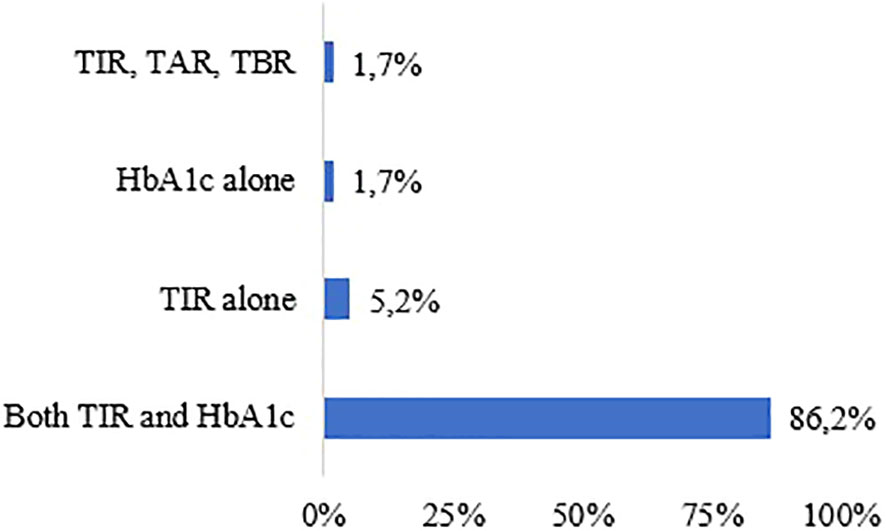
Preferred Parameters to Measure Glycemic Control.

While 86·2% of the physicians stated that they place emphasis on both TIR and lab HbA1c parameters for glycemic control of diabetic patients using MDI, 5·2% and 1·7% mentioned only TIR and only HbA1c results, respectively. Additionally, a minority of participants (1·7%) expressed the importance of considering TIR, TAR, and TBR (**Figure 3**).

Satisfaction with TIR that was achieved in diabetic patients treated with MDI+BGM was expressed by 31% of physicians, while 27·5% remained neutral and 41·3% were dissatisfied. The majority of participants (77·5% and 82·7%) reported satisfaction with the achieved TIR in MDI+isCGM and MDI+CGM treatments, respectively. Participants reported satisfaction with HbA1c results in the following proportions: 32·7% with MDI+BGM treatment, 63·7% with MDI+isCGM treatment, and 68·9% with MDI+CGM treatment.

According to the study findings, 74·1% of physicians believed that Real-Time CGM (rtCGM) treatment was satisfactory for achieving TIR targets in diabetic patients. A smaller proportion (10·3%) considered the treatment to be insufficient, while 15·5% remained uncertain. The majority of physicians (79·3%) believed that rtCGM treatment was adequate for achieving their HbA1c goals, with 15·5% remaining neutral and 5·1% considering the treatment to be insufficient (**Table 2**).

**Table 2:** Use of TIR or HbA1c satisfaction rate by type of treatment.

	Satisfaction by use of TIR			Satisfaction by use of HbA1c		
	MDI+BGM	MDI+isCGM	MDI+CGM	MDI+BGM	MDI+isCGM	MDI+CGM
Satisfaction Level	31·3%	77·6%	82·8%	32·8%	63·8%	82·8%
Additional glucose data beyond BGM alone	22·4%	16·6%	–	29·8%	33·3%	–
Data on missed/late insulin doses	24·1%	25·0%	25·0%	22·8%	20·0%	28·5%
Prevention of Insulin stacking	17·2%	25·0%	25·0%	19·3%	13·3%	28·5%
More frequent blood glucose testing per day	18·9%	–	–	–	–	–
Data on miscalculated insulin doses	15·5%	33·3%	25·0%	26·3%	33·3%	42·8%
Not using additional features of CGM	–	–	25·0%	–	–	–

##### Open discussion

During the discussion sessions, physicians from the Middle East region raised concerns about the potential for misleading results with HbA1c, particularly among pregnant women or patients with Down syndrome or hemoglobinopathies. As such, they suggested that TIR and HbA1c be assessed in conjunction. Physicians from Eastern Europe, on the other hand, expressed less satisfaction with HbA1c results when using MDI+isCGM and MDI+CGM treatments. Furthermore, some physicians noted that HbA1c might not be necessary if CGM was utilized.

According to the physicians in the Middle East region, the proportion of patients achieving the target TIR was relatively low (20-30%) despite the analysis results indicating that patients treated with CGM were able to achieve their TIR targets. Similarly, physicians from Eastern Europe reported that the rate of patients using isCGM who achieved the TIR target was approximately 30%. They also noted that while HbA1c results in patients using isCGM were promising during the first two weeks, there were some misleading results in the subsequent period.

##### Questionnaire: Attitudes and concepts about barriers & drivers to achieve glycemic targets

According to the responses, it is perceived that an average of 44% of diabetic patients using MDI adhered to carbohydrate counting. This rate was higher in the pediatric population than in adults (51% vs 36%). Among diabetic patients using MDI, the frequency of using the sensor per month is 52% and it is similar in in both pediatric & adult populations (54% vs 52%). The study findings revealed that 24% of physicians reported that diabetic patients using MDI could potentially miss two or more doses per week. According to the results, the rate of missed doses among patients using MDI was found to be 22% for adults and 26% for pediatric patients. Furthermore, the study revealed that 32% of patients with diabetes using MDI were at risk of miscalculating their insulin dose. Consequently, the proportion of patients using MDI who may experience level 2 and level 3 hypoglycemia due to insulin accumulation was estimated to be 22% (**Table 3**).

**Table 3:** Problems experienced in diabetic patients using MDI.

	Pediatric	Adult	Overall
Concordance with carb counting	51%	36%	44%
Sensor utilization	54%	52%	52%
Missing ≥2 Bolus doses per week	26%	22%	24%
Miscalculating the insulin dosage	34%	30%	32%
Level 2 and level 3 hypoglycemia (<54 mg/dl)	22%	22%	22%

##### Open discussion

In the discussion session, physicians from the Middle East region stated that they found these rates to be accurate and that their patients missed doses and calculated the dosage wrongfully almost every day. It was conceded that patients, pay little attention to what they eat in a day, and they believed that missed doses are the major problem according to their clinical practice. In addition, all physicians agree that carb counting is more prevalent in the pediatric population with T1D. During the discussion sessions, physicians from the Eastern Europe region noted that the use of sensors appeared to be more prevalent, possibly due to geographical and financial factors.

##### Questionnaire: Attitudes and concepts with regard to the value and importance of Smart MDI System

It was found that a significant majority (93%) of participants agreed that patients with diabetes using MDI would appreciate the additional clinical benefits of the Smart MDI System. Additionally, participants believed that the Smart MDI System would offer greater clinical advantages than MDI+BGM (93%), MDI+isCGM (86%), and MDI+CGM (81%) applications (**Table 4**). Participants evaluated clinical benefits of Smart MDI System provided by the system on a 5-point Likert scale. Depending on the evaluation, the pre-elimination of errors in insulin dose according to Likert scale was rated as 4·28/5, the complete picture of diabetes management as 4·18/5, the elimination of missed doses as 4·09/5, insulin stacking as 4·05/5 and the simplification of meal management (carb count) as 3·93/5 (**Table 5**).

**Table 4:** Additional clinical benefit of the smart MDI system for diabetic patients using MDI.

	Agree & Strongly Agree	Neutral	Disagree & Strongly Disagree
General additional clinical benefits of Smart MDI System	92·9%	5·2%	1·7%
Smart MDI System vs MDI+BGM	92·9%	5·2%	1·7%
Smart MDI System vs MDI+isCGM	85·9%	10·5%	3·5%
Smart MDI System vs MDI+CGM	80·7%	15·7%	3·5%

**Table 5:** Evaluation of clinical benefits and importance of the smart MDI system: results from a 5-point likert scale.

	1	2	3	4	5	Mean
Clinical Benefits						
Elimination of insulin dosage mistakes	1 (1·75%)	1 (1·75%)	4 (7·02%)	26 (45·61%)	25 (43·86%)	4·28
Full picture of diabetes management	1 (1·75%)	1 (1·75%)	9 (16·36%)	20 (36·36%)	24 (43·64%)	4·18
Eliminating missed doses	3 (5·26%)	0 (0·00%)	11 (19·30%)	18 (31·58%)	25 (43·86%)	4·09
Insulin stacking	1 (1·75%)	3 (5·26%)	10 (17·54%)	21 (36·84%)	22 (38·60%)	4·05
Simplifying meal management	2 (3·51%)	3 (5·26%)	11 (19·30%)	22 (38·60%)	19 (33·33%)	3·93
Important Patient Groups						
Diabetic patients who are suitable for AHCL but cannot access it due to affordability	0 (0·00%)	1 (1·79%)	10 (17·86%)	14 (25·00%)	31 (55·36%)	4·34
Diabetic patients already on Basal: Bolus on either BGM, CGM or isCGM with unsatisfactory clinical outcomes	0 (0·00%)	3 (5·36%)	12 (21·43%)	24 (42·86%)	17 (30·36%)	4·00
Diabetic patients who are suitable for AHCL but unfavored by the user	2 (3·57%)	4 (7·14%)	13 (23·21%)	19 (33·93%)	18 (32·14%)	3·84
Diabetic patients with Basal: Bolus initiation irrespective of glucose monitoring technology	2 (3·57%)	4 (7·14%)	12 (21·43%)	23 (41·07%)	15 (26·79%)	3·80

The importance of a Smart MDI System for diabetic patients using MDI has been also evaluated on a 5-point Likert scale based on the categories. According to the analysis results, Smart MDI System has the highest level of importance for diabetic patients who are suitable for Advanced Hybrid Closed Loop System (AHCL) but cannot access it due to affordability (4·34/5). Other diabetic patients categories where a Smart MDI System is important were mentioned as: a) bolus on either BGM, CGM or isCGM with unsatisfactory clinical outcomes (4·00/5); b) unfavored by the user (3·84/5); c) bolus initiation irrespective of glucose monitoring technology (3·80/5) (**Table 5**).

During the study, the possible acceptance and usage of a Smart MDI System among diabetic patients using MDI were assessed. Results showed that 49% of the participants thought that diabetic patients using MDI would be willing to use a Smart MDI System. Majority of physicians (96%) believed that diabetic patients who start the Smart MDI System would continue using it for more than 6 months. The study examined physicians’ views on the appropriate pricing for a Smart MDI System compared to the current CGM prices in their respective countries. Results indicated that 50% of the physicians thought that the Smart MDI System should be priced 10 to 30% higher than the current CGM prices, while 27·5% believed that the price should be 31 to 50% higher and 22·5% believed that the pricing should be similar.

##### Open discussion

During the discussion session, physicians, particularly from the Eastern Europe region, emphasized that the use of a Smart MDI System is contingent on patient preference and cost. The anticipated level of adoption by patients was set to exceed 49%. In contrast, physicians from the Middle East expressed their view that patients aged 50-60 may not be inclined to use the new technology.

## Discussions

The present study highlights that physicians are generally supportive of utilizing new technology. However, during open discussions, concerns were raised due to their limited practical experience with Smart MDI Systems. Additionally, there are concerns about accessing diabetic patients’ phone, which could hinder optimal use of the technology. The questionnaires and the open discussion revealed the expectation that the Smart MDI technology provides better control, primarily by identifying missed boluses, while expressing concerns on the use of the technology by teenagers and children, who might forget the device and be reluctant to use in public, and by the older population, who might be challenged by the technology.

This study revealed gaps in the knowledge and experience of CGM in clinical use by physicians from the Middle East region. This gap may translate into a lower rate of use by patients. Physicians mentioned that online training should be given to patients and healthcare professionals about how to use the device, settings, and solutions to possible problems.

Another important barrier expressed, notably by Middle East and African region physicians, is the affordability. In contrast to the views of physicians from the Middle East, physicians from the Central-Eastern Europe region pointed out that the insurance systems in their countries cover the cost of new technologies, including Smart MDI System, subject to meeting certain conditions, and that reimbursement is available.

Based on the results and discussions, it is clear that the main hurdles to overcome are the access of the new technologies and the lack of experience of HCPs. Physicians should raise awareness and seek to negotiate with policy makers to include new technology in reimbursement, taking into account the perceived clinical utility of the new technology and the improved quality of life for patients.

### Limitations

This study is based on a technique to assess attitudes and perception therefore cannot be used for quantitative statistical analysis. Since the participants were predominantly specialized physicians attending The Diabetes Innovation Summit 2023, primary care physicians, who are also integral to diabetes management, were not included in the sample. Additionally, the survey did not collect supplementary data beyond the questions presented in the questionnaire, as outlined in the **Supplementary Materials**. Therefore, further studies are needed to address these limitations and provide a more comprehensive understanding.

## Data Availability

The raw data supporting the conclusions of this article will be made available by the authors, without undue reservation.
