# Supplementary material for: Clinical perspective on innovative insulin delivery technologies in diabetes management
Source: Front Endocrinol (Lausanne). 2024 Oct 1;15:1308319. doi: 10.3389/fendo.2024.1308319 (PMC11473347; doi:10.3389/fendo.2024.1308319)
Supplement: Supplementary file 1 [file DataSheet1.pdf]

PRESENTATION QUESTIONS

1. You are a(an)
2. N/AWhat is the estimated percentage of your PwD who are on MDI (with respect to your specialty, considering the size of T1D or T2D in your practice)?
3. What is the estimated percentage of those PwD on MDI who are using BGM?
4. What is the estimated percentage of those PwD on MDI who are using isCGM?
5. What is the estimated percentage of those PwD on MDI who are using CGM?
6. What is your preferred parameter to evaluate glycemic control for PwD on MDI?
7. Are you satisfied with the Time in Range of PwD treated with MDI + BGM?
   7.1. If your answer is "strongly disagree / disagree", what should be improved in treating PwD on MDI + BGM?
8. Are you satisfied with the Time in Range of PwD treated with MDI + isCGM?
   8.1. If your answer is "strongly disagree / disagree", what should be improved in treating PwD on MDI + isCGM?
9. Are you satisfied with Time in Range of PwD treated with MDI + CGM?
   9.1. If your answer is "strongly disagree / disagree", what should be improved in treating PwD on MDI + CGM?
10. Are you satisfied with the HbA1c of PwD treated with MDI + BGM?
    10.1. If your answer is "strongly disagree / disagree", what should be improved in treating PwD on MDI + BGM
11. Are you satisfied with the HbA1c (Lab test) of PwD treated with MDI + isCGM?
    11.1. If your answer is "strongly disagree / disagree", what should be improved in treating PwD on MDI plus isCGM?
12. Are you satisfied with the HbA1c of PwD treated with MDI + CGM?

13.1. If your answer is "strongly disagree / disagree", what should be improved in treating PwD on MDI plus CGM?

14. Do you think that Real-Time CGM is sufficient for your PwD on MDI to reach HbA1c goals?

15. Do you think that Real-Time CGM is sufficient for your PwD on MDI to reach Time in Range target (>70% of time spent between 70-180 mg/dl)?

16. What is the estimated % of your PwD on MDI who are adhering to carb counting?

17. Out of PwD using the CGM, what is the % of Sensor utilization per month?

18. What is the estimated % of your PwD on MDI who may miss ≥ 2 Bolus doses per week?

19. What is the estimated percentage of your PwD on MDI who may miscalculate the insulin dosage?

20. What is the estimated percentage of your PwD on MDI who may suffer from level 2 and level 3 hypoglycemia (< 54 mg/dl) due to insulin stacking?

21. Could Smart MDI System (Smart InPen + G4S) offer additional clinical benefits to your PwD on MDI in managing their diabetes?

22. Could Smart MDI System (Smart InPen + G4S) provide additional clinical advantages versus regular (MDI + BGM) for your PwD on MDI?

23. Could Smart MDI System (Smart InPen + G4S) provide additional clinical advantages versus regular (MDI + isCGM) for your PwD on MDI?

24. Could Smart MDI System (Smart InPen + G4S) provide additional clinical advantages versus regular (MDI + CGM) for your PwD on MDI?

25. In which areas do you think Smart MDI System (Smart InPen + G4S) could offer the most benefits for PwD on MDI? Rate using 1 to 5 – Elimination of insulin dosage mistakes

25.1. In which areas do you think Smart MDI System (Smart InPen + G4S) could offer the most benefits for PwD on MDI? Rate using 1 to 5 – Simplifying meal management (carbohydrate counting)

25.2. In which areas do you think Smart MDI System (Smart InPen + G4S) could offer the most benefits for PwD on MDI? Rate using 1 to 5 – Eliminating missed doses

25.3. In which areas do you think Smart MDI System (Smart InPen + G4S) could offer the most benefits for PwD on MDI? Rate using 1 to 5) – Insulin stacking

25.4. In which areas do you think Smart MDI System (Smart InPen + G4S) could offer the most benefits for PwD on MDI? Rate using 1 to 5 – Full picture of Diabetes management

26. On a scale from 1 to 5, how would you value the importance of Smart MDI System (Smart InPen plus G4S) in each of the following category of PwD on MDI? – PwD with Basal: Bolus initiation irrespective of glucose monitoring technology

26.1. On a scale from 1 to 5, how would you value the importance of Smart MDI System (Smart InPen plus G4S) in each of the following category of PwD on MDI? – PwD with Basal: Bolus on either BGM, CGM or isCGM with unsatisfactory clinical outcomes

26.2. On a scale from 1 to 5, how would you value the importance of Smart MDI System (Smart InPen plus G4S) in each of the following category of PwD on MDI? – PwD who are eligible to advanced hybrid closed loop (AHCL) but can't access it due to affordability

26.3. On a scale from 1 to 5, how would you value the importance of Smart MDI System (Smart InPen plus G4S) in each of the following category of PwD on MDI? – PwD who are eligible to advanced hybrid closed loop (AHCL) but unfavored by the user

27. What percentage of PwD on MDI would accept to start using the Smart MDI System (Smart InPen + G4S)?

28. What is the likelihood of PwD starting on Smart MDI would continue using the system for 6 months?
